# Long-lived self-renewing bone marrow-derived macrophages displace embryo-derived cells to inhabit adult serous cavities

**DOI:** 10.1038/ncomms11852

**Published:** 2016-06-13

**Authors:** Calum C. Bain, Catherine A. Hawley, Hannah Garner, Charlotte L. Scott, Anika Schridde, Nicholas J. Steers, Matthias Mack, Anagha Joshi, Martin Guilliams, Allan Mc I. Mowat, Frederic Geissmann, Stephen J. Jenkins

**Affiliations:** 1 The University of Edinburgh/MRC Centre for Inflammation Research, Queens Medical Research Institute, 47 Little France Crescent, Edinburgh EH16 4TJ, UK; 2 Centre for Molecular and Cellular Biology of Inflammation (CMCBI), New Hunt's House, King's College London, Great Maze Pond, London SE1 1UL, UK; 3 Unit of Immunoregulation and Mucosal Immunology, VIB Inflammation Research Center, Ghent 9000, Belgium; 4 Department of Biomedical Molecular Biology, Ghent University, Ghent 9000, Belgium; 5 Institute of Infection, Immunity and Inflammation, University of Glasgow, Sir Graeme Davies Building, 120 University Place, Glasgow G12 8TA, UK; 6 Institute of Molecular Medicine, RWTH University, 52074 Aachen, Germany; 7 Department of Internal Medicine - Nephrology University Hospital Regensburg, Regensburg 93042, Germany; 8 The Roslin Institute and Royal (Dick) School of Veterinary Studies, Easter Bush Campus, University of Edinburgh, Midlothian EH25 9RG, UK; 9 Immunology Program, Memorial Sloan Kettering Cancer Center, 417 East 68th Street, New York, New York 10065, USA; 10 Weill Cornell Graduate School of Medical Sciences, New York, New York 10065, USA

## Abstract

Peritoneal macrophages are one of the most studied macrophage populations in the body, yet the composition, developmental origin and mechanisms governing the maintenance of this compartment are controversial. Here we show resident F4/80^hi^GATA6^+^ macrophages are long-lived, undergo non-stochastic self-renewal and retain cells of embryonic origin for at least 4 months in mice. However, Ly6C^+^ monocytes constitutively enter the peritoneal cavity in a CCR2-dependent manner, where they mature into short-lived F4/80^lo^MHCII^+^ cells that act, in part, as precursors of F4/80^hi^GATA6^+^ macrophages. Notably, monocyte-derived F4/80^hi^ macrophages eventually displace the embryonic population with age in a process that is highly gender dependent and not due to proliferative exhaustion of the incumbent embryonic population, despite the greater proliferative activity of newly recruited cells. Furthermore, although monocyte-derived cells acquire key characteristics of the embryonic population, expression of Tim4 was impaired, leading to cumulative changes in the population with age.

## Abstract (web summary)

Understanding the heterogeneity of peritoneal macrophages is hampered by controversy over their origin and homeostasis. Here the authors show the embryonic F4/80hi population is replaced over time by self-renewing bone marrow-derived cells transitioning from F4/80lo to F4/80hi in adult mice, and that such turnover is more rapid in male mice.

## Graphical abstract

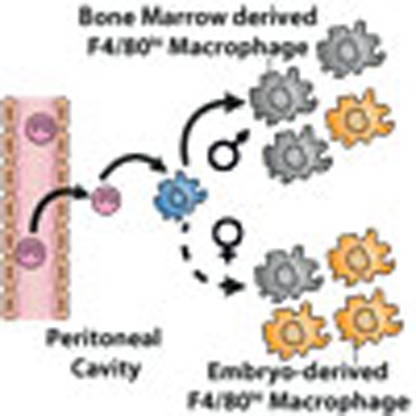

The myeloid cell compartment of steady-state tissues is highly heterogeneous, comprising cells of diverse origin and function. These cells include mononuclear phagocytes, such as macrophages and dendritic cells (DC), which have vital roles in tissue homoeostasis, protective immunity and wound healing and tissue remodelling after injury. Early studies suggest that macrophages are part of a discrete mononuclear phagocyte system, continually replenished by circulating blood monocytes^1^,^2^. More recent studies propose that tissue macrophages derive from embryonic progenitors that seed tissues during development^3^,^4^, and are subsequently maintained autonomously from conventional haematopoiesis by self-renewal and/or longevity^5^,^6^,^7^. Whether all macrophages are equally capable of self-renewal or if ‘progenitor cells' exist within the population is unclear. Whereas proliferation within the alveolar macrophage compartment is reported to occur stochastically^6^, clonal self-renewal among Langerhans cells suggests the presence of local progenitors^8^. However, the paradigm has now shifted once again, as a crucial role for adult monocytes in the replenishment of resident macrophages has been confirmed in the gut wall^9^, the dermis^10^ and the heart^11^,^12^. Notably, in these tissues, reliance on blood monocytes for macrophage replenishment increases with age, parallels the loss of macrophages deriving from embryonic sources and seems to accompany loss of proliferation within the embryonic population.

The peritoneal cavity is home to a complex mix of immune cells with important functions in monitoring visceral organs and related mesothelium^13^, including phagocytes marked by high expression of F4/80 (F4/80^hi^) that share a common gene signature with many tissue macrophages^14^. An additional, less abundant and phenotypically distinct group of phagocytes is present in the peritoneal cavity, distinguished by lower F4/80 (F4/80^lo^) and higher MHCII expression and sometimes referred to as small peritoneal macrophages (SPM)^15^. In contrast to F4/80^hi^ resident peritoneal macrophages^13^,^16^,^17^, relatively little is known about the functionality of the F4/80^lo^MHCII^+^ cells, probably due to lack of consensus on which cells comprise this population. For instance, some reports consider all F4/80^lo^CD11b^+^MHCII^+^ cells as genuine macrophages^13^,^18^, whereas others suggest this population contains CD11c^+^MHCII^+^ DC and exclude CD11c^+^ cells when identifying SPM^15^,^19^,^20^. In another approach, CSF1R expression and MHCII were used to identify F4/80^lo^ macrophages^14^,^20^,^21^; however, these studies did not assess the possibility for DC to express CSF1R. Thus, the identity of F4/80^lo^ cells in the peritoneal cavity remains unclear.

The contribution of monocytes to the replenishment of peritoneal macrophage populations also remains controversial. Although reduced in number in monocytopenic *Ccr2*^−/−^ mice^13^, F4/80^lo^ macrophages are not labelled in fate mapping studies using tamoxifen-inducible *Cx3cr1*^creER^*Rosa26*^YFP^ reporter mice to track monocytes^5^. F4/80^hi^ macrophages appear unaffected by *Ccr2* deficiency^13^, fail to exchange to any great extent in parabiotic mice^6^, and do not label in *Cx3cr1*^creER^-driven reporter mice^5^, suggesting that they exist independent of blood monocytes. Furthermore, significant proliferation is evident within this population^22^,^23^,^24^. However, *Kit-*driven fate mapping suggests that peritoneal F4/80^hi^ macrophages have a short half-life, being replaced more rapidly than colonic macrophages^25^. Whether F4/80^hi^ and F4/80^lo^ populations represent independent subsets of macrophages, or if they are related developmentally, is also unclear.

Here, we use multiple fate mapping approaches and non-invasive methods of tracking proliferation history to interrogate heterogeneity, origin and self-renewal capacity of peritoneal macrophages. We show that although seeded by embryonic precursors, the F4/80^hi^ macrophage compartment is increasingly replaced, with age, by bone marrow-derived monocytes at a rate which, unlike for most other tissue macrophages, is gender specific, perhaps reconciling the discrepant findings from previous studies^5^,^6^,^13^,^25^. Furthermore, we demonstrate that F4/80^lo^ macrophages are rapidly replenished by Ly6C^+^ monocytes recruited via a CCR2-dependent mechanism, and that these act, in part, as short-lived intermediaries between newly arrived monocytes and fully mature F4/80^hi^ macrophages. We also reveal that there is considerable heterogeneity in the proliferative activity of the F4/80^hi^ macrophage compartment, and contrary to current dogma, demonstrate that newly monocyte-derived F4/80^hi^ macrophages possess the highest level of proliferative activity.

## Results

### Phenotypic profiling of cavity myeloid populations

**Figure 1:**
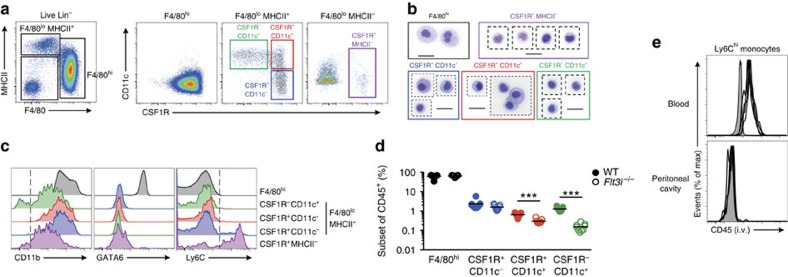
Phenotypic characterization of peritoneal myeloid cells. (**a**) Expression of F4/80 and MHCII by live CD45^+^Lin^−^ (CD3, CD19, Ly6G, SiglecF) peritoneal exudate cells (PEC) obtained from naïve adult WT mice (left panel) and expression of CD11c and CSF1R by F4/80^hi^, F4/80^lo^MHCII^+^ and F4/80^lo^MHCII^−^ cells (right panel). Data are representative of at least 20 independent experiments. (**b**) Morphological appearance of F4/80^hi^, F4/80^lo^MHCII^+^ subsets and F4/80^lo^MHCII^−^ cells purified from naive adult WT mice. Scale bar, 20 μm. Images are from one experiment performed. (**c**) Expression of CD11b, GATA6 and Ly6C by PEC myeloid subsets as gated in **a**. Histograms are representative of at least six (GATA6) or 20 (CD11b and Ly6C) independent experiments. (**d**) Frequency among CD45^+^ leukocytes of F4/80^hi^ macrophages and CD11c/CSF1R-defined F4/80^lo^MHCII^+^ subsets in PEC of naive 8-week old WT (*n*=9) or *Flt3l*^−/−^ (*n*=9) mice. Data are pooled from two independent experiments. Each symbol represents an individual animal and horizontal bars represent the mean. ***P<0.0001 determined by Student's *t*-test followed by Holm-Sidak correction. (**e**) Expression of CD45 by Ly6C^+^ monocytes in blood and peritoneal cavity of WT mice administered anti-CD45 i.v. 2 min before necropsy. Each line represents an individual mouse (*n*=4) and shaded histograms represent uninjected control mice. Data representative of four independent experiments.

To resolve outstanding questions on peritoneal macrophage origins, we rigorously re-examined the composition of the myeloid compartment among peritoneal exudate cells (PEC). Among CD3^−^CD19^−^Ly6G^−^SiglecF^−^ cells, two populations of F4/80^+^ cells were present as described previously^14^,^15^,^19^,^21^; F4/80^hi^ cells that expressed little MHCII, and F4/80^lo^ cells that were mostly MHCII^+^ (Fig. 1a; Supplementary Fig. 1). Given the inconsistencies in use of CD11c or CSF1R to define PEC of dendritic cell and macrophage lineages, respectively, we examined expression of these markers in combination. Consistent with previous work^19^, F4/80^hi^ cells were homogeneously CSF1R^+^CD11c^−^, dominated the myeloid compartment and displayed typical macrophage morphology (Fig. 1a,b). In contrast, the F4/80^lo^MHCII^+^ population was heterogeneous, comprising CSF1R^+^CD11c^–^ cells, CSF1R^–^CD11c^+^ cells but also those expressing both markers (Fig. 1a). CSF1R^+^CD11c^–^ and CSF1R^+^CD11c^+^ cells uniformly expressed CD11b and while most CSF1R^–^CD11c^+^ cells expressed CD11b (Fig. 1c), a small fraction was CD11b^−^ but expressed both CD103 and XCR1 (Supplementary Fig. 1). These CSF1R^–^CD11c^+^ cells were highly dependent on Flt3L for their development (Fig. 1d) and expressed the cDC-specific marker CD272 (Supplementary Fig. 1)^26^. Thus, CSF1R^–^CD11c^+^ cells represent *bona fide* DC and mirror the subsets of conventional DC (cDC) in other tissues^27^. Notably, the CSF1R^+^CD11c^+^ population was also reduced in *Flt3l*^−/−^ mice (Fig. 1d), suggesting it may also contain cDC and that CSF1R cannot be used alone as a *de facto* marker of PEC macrophages.

We also identified F4/80^lo^MHCII^–^CSF1R^+^ cells that had the morphological appearance of monocytes (Fig. 1a,b), mostly expressed the monocyte marker Ly6C (Fig. 1c) and which were genuine inhabitants of the peritoneal cavity, as they did not label with anti-CD45 mAb injected i.v. before necropsy, a procedure that labels >90% of circulating blood monocytes (Fig. 1e)^28^. Thus the steady-state peritoneal cavity contains F4/80^hi^ macrophages and Ly6C^+^ monocytes, as well as MHCII^+^ cells comprised of DC (CSF1R^−^CD11c^+^), F4/80^lo^CSF1R^+^CD11c^–^ cells and a potentially heterogeneous population of F4/80^lo^CSF1R^+^CD11c^+^ cells.

### Diverse proliferative activity among F4/80^hi^ macrophages

**Figure 2:**
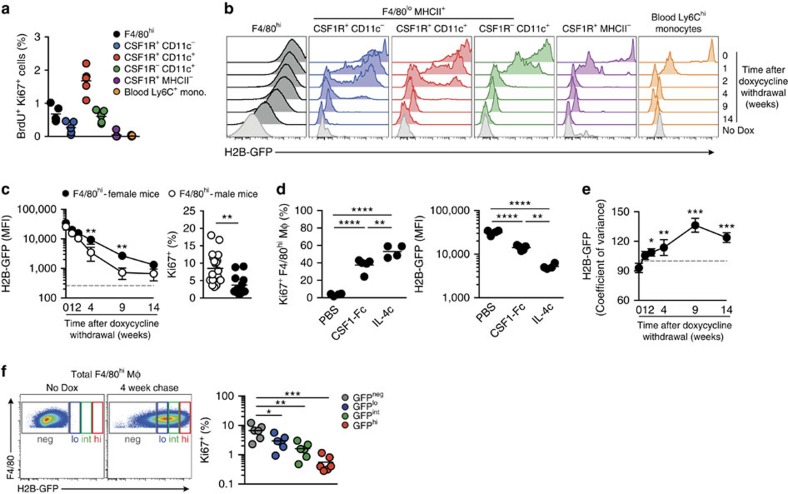
Heterogeneity in proliferative activity of F4/80^hi^ macrophages. (**a**) Frequency of BrdU^+^Ki67^+^ cells amongst peritoneal F4/80^hi^ macrophages, Ly6C^+^ monocytes and CD11c/CSF1R-defined F4/80^lo^MHCII^+^ subsets, and Ly6C^+^ blood monocytes from 8-week old female WT mice administered 1 mg BrdU s.c. 2 h before necropsy. Data are from one of two independent experiments with 5 mice per group. (**b-f**) H2B-GFP mice were administered doxycycline diet for 2 weeks before the expression of H2B-GFP by PEC subsets was assessed by flow cytometry. (**b**) Expression of H2B-GFP fusion protein by peritoneal F4/80^hi^ macrophages, Ly6C^+^ monocytes and CD11c/CSF1R-defined F4/80^lo^ MHCII^+^ subsets, and Ly6C^+^ blood monocytes from H2B-GFP mice 0, 1, 2, 4, 9 and 14 weeks following the cessation of doxycycline administration. Data are from one experiment of two performed with 3 (0, 1, 2, 4 and 9 weeks) or 4 (14 weeks) mice per time point. Light grey histograms represent the background fluorescence of each cell population obtained from mice that did not receive doxycycline (‘No Dox'). (**c**) Mean fluorescence intensity (MFI) of H2B-GFP expression (left) and Ki67 expression by F4/80^hi^ macrophages from the female (solid circles) and male (open circles) H2B-GFP mice as in **b**. One-way ANOVA followed by Tukey's multiple comparison test (H2B-GFP MFI) and upaired Student's *t*-test (Ki67; **P<0.01). (**d**) Expression of Ki67 (left) and MFI of H2B-GFP expression (right) by PEC F4/80^hi^ macrophages from mice administered CSF1-Fc, IL-4c or vehicle control at day 1 and day 3 post-cessation of doxycycline and analysed 1 day later. Data from one of two independent experiments with 4 (PBS, IL-4c) or 5 (CSF1-Fc) mice. One-way ANOVA followed by Tukey's multiple comparison test. **P<0.01, ****P<0.0001. (**e**) Co-efficient of variance of H2B-GFP expression by F4/80^hi^ macrophages from the peritoneum of mice in **b**. One-way ANOVA followed by Tukey's multiple comparison test. *P<0.05, **P<0.01, ***P<0.001. (**f**) Gating strategy used to define GFP^hi^, GFP^int^, GFP^lo^ and GFP^–^ F4/80^hi^ macrophages from H2B-GFP mice administered doxycycline diet for 2 weeks and ‘chased' for 4 weeks (left) and expression of Ki67 by the GFP-defined fractions of, F4/80^hi^ macrophages. One-way ANOVA followed by Tukey's multiple comparison test. *P<0.05, **P<0.01, ***P<0.001. Each symbol represents an individual animal and horizontal bar is the mean. Data represent 6 mice and are pooled from two independent experiments.

Peritoneal F4/80^hi^ macrophages are reported to exist autonomously of blood monocytes^5^,^6^, suggesting that *in situ* self-renewal might be the principal mechanism for their homoeostatic maintenance. Indeed, as previously reported^16^,^23^,^29^, low-level proliferation measured by BrdU incorporation and Ki67 expression was detectable in F4/80^hi^ macrophages (Fig. 2a). However, whether proliferation is purely stochastic or involves a dedicated local macrophage progenitor remains poorly understood. Indeed, heterogeneity in the ability of resident peritoneal macrophages to proliferate in response to CSF1 has been suggested by recent single-cell gene expression analyses^30^. Thus, to assess the diversity in proliferative activity among peritoneal F4/80^hi^ macrophages, we utilized mice that allow transient but ubiquitous, doxycycline-inducible expression of a histone 2B (H2B)-GFP fusion protein that can distinguish slowly dividing stem cells in bone marrow (BM), which remain H2B-GFP^+^ for over 1 year, from their more rapidly dividing (GFP^–^) progeny^31^. Administration of doxycycline for 2 weeks led to full and uniform H2B-GFP labelling of F4/80^hi^ macrophages, as well as F4/80^lo^ populations (Fig. 2b) and monocytes in blood (Fig. 2b). Ly6C^+^ blood and cavity monocytes rapidly lost H2B-GFP (Fig. 2b) despite not actively proliferating (Fig. 2a), consistent with their short half-life and derivation from rapidly proliferating BM progenitors^31^. Loss of label by F4/80^lo^MHCII^+^ subsets occurred within ∼4 weeks but in a bimodal manner (Fig. 2b), suggestive of both loss due to proliferation *in situ* (Fig. 2a) and potential inclusion of GFP^–^ BM-derived cells. In contrast, F4/80^hi^ macrophages lost H2B-GFP expression gradually and relatively uniformly over a 14-week ‘chase' period, confirming the relative autonomy of this population and indicating appreciable but low-level self-renewal across the entire population during this period. That H2B-GFP loss tracked proliferation history was supported by accelerated loss of H2B-GFP expression by PEC F4/80^hi^ macrophages from male mice compared with those from female mice that corresponded with the higher frequency of proliferating Ki67^+^ cells detectable in males (Fig. 2c). Furthermore, administration of IL-4c or CSF1-Fc, which are known to drive proliferation of peritoneal macrophages^23^,^24^, led to an enhanced rate of GFP loss that reflected the level of proliferation induced (Fig. 2d). Importantly, we did not detect any quiescent F4/80^hi^ macrophages retaining high levels of H2B-GFP that could represent a local precursor (Fig. 2b). Nevertheless, the variance around the mean of H2B-GFP expression by the F4/80^hi^ macrophage population increased progressively following doxycycline withdrawal (Fig. 2e), suggesting some diversity in proliferative activity within the population. Notably, comparison of proliferation history with proliferative activity revealed that cells retaining the highest levels of GFP also contained the fewest cycling Ki67^+^ cells, while GFP^–/lo^ F4/80^hi^ macrophages that had undergone most proliferation continued to undergo greater proliferation (Fig. 2f). These data support a model of non-stochastic proliferation within the F4/80^hi^ macrophage population in which certain cells inherently undergo higher levels of proliferation. Taken together these data suggest that it is unlikely that quiescent local progenitors exist amongst the F4/80^hi^ macrophage population in the serous cavities, despite heterogeneity in proliferative activity within this compartment.

### CCR2-dependent precursors replenish F4/80^hi^ macrophages

**Figure 3:**
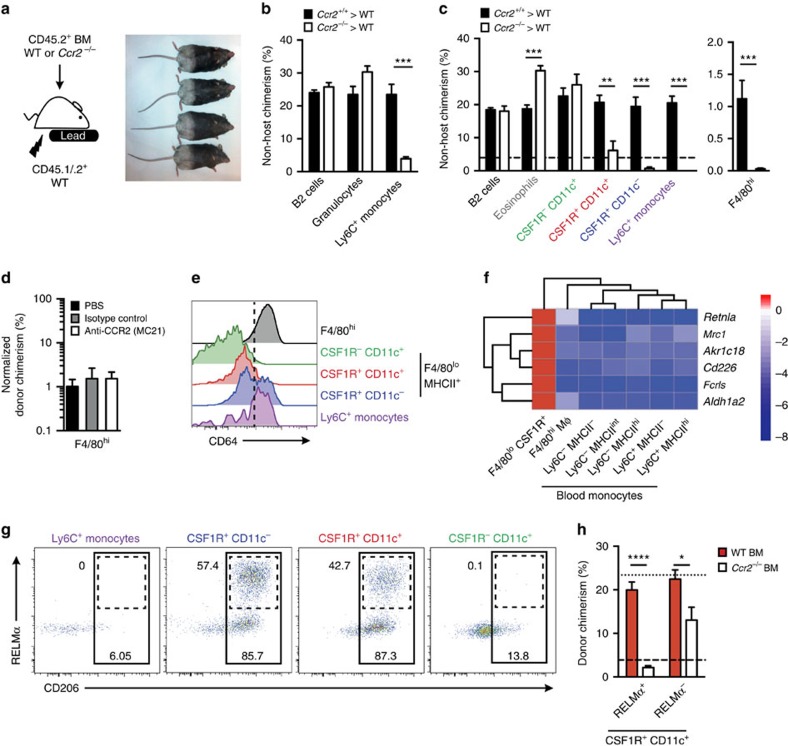
Monocytes recruited via CCR2 replenish CSF1R^+^ but not CSF1R^−^ F4/80^lo^ cells. (**a**) Experimental scheme and appearance of WT>WT tissue-protected BM chimeric mice 36 weeks after irradiation and reconstitution, highlighting the area exposed to radiation. (**b**) Non-host chimerism amongst B2 B cells, total granulocytes and Ly6C^+^ monocytes in the blood of tissue-protected BM chimeric mice 8 weeks after irradiation and reconstitution with *Ccr2*^+/+^ (black bars) or *Ccr2*^−/−^ (white bars) BM. (**c**) Non-host chimerism amongst B2 B cells, eosinophils, F4/80^hi^ macrophages, Ly6C^+^ monocytes and CD11c/CSF1R-defined F4/80^lo^MHCII^+^ subsets in the PEC of mice in **b**. Bars in **b** and **c** represent mean+s.e.m. and data are from one of four experiments performed. Dashed line represents chimerism in Ly6C^+^ blood monocytes. Where shown, **P<0.005, ***P<0.001 determined by Student's *t*-test followed by Holm-Sidak correction. (**d**) The mean non-host chimerism amongst peritoneal F4/80^hi^ macrophages of tissue-protected BM chimeric mice that received anti-CCR2, isotype control or PBS at the point of reconstitution. Bars represent the mean+s.d. and are from one experiment with 4 (PBS, isotype control) or 5 (anti-CCR2) mice. (**e**) Representative expression of CD64 by peritoneal F4/80^hi^ macrophages, CD11c/CSF1R-defined F4/80^lo^MHCII^+^ subsets and Ly6C^+^ monocytes from WT mice. Representative histograms from one of three independent experiments performed. (**f**) Heatmap highlighting the genes specifically enriched in PEC F4/80^lo^MHCII^+^ cells compared with F4/80^hi^ macrophages and subsets of blood monocytes. Only the six most highly differentially expressed genes shown. (**g**) Expression of RELMα and CD206 by Ly6C^+^ monocytes and CD11c/CSF1R-defined F4/80^lo^MHCII^+^ subsets from the peritoneal cavity of 8-week old WT mice. Gates were set using FMO controls and staining is from one representative experiment of two performed. (**h**) Non-host chimerism amongst RELMα^+^ and RELMα^−^ CD11c^+^CSF1R^+^ PEC of tissue-protected BM chimeric mice reconstituted with *Ccr2*^+/+^ (red bars) or *Ccr2*^−/−^ (white bars) BM. *P<0.05, ****P<0.0001 determined by Student's *t*-test followed by Holm-Sidak correction. Bars represent the mean+s.e.m. and are from one experiment representative of two performed with 5 mice per group. Dotted and dashed lines represents chimerism in Ly6C^+^ blood monocytes in recipients of WT and *Ccr2*^−/−^ BM respectively.

The lack of detectable label-retaining F4/80^hi^ macrophages in our H2B-GFP studies prompted us to re-examine the long-term autonomy of this population. To assess the contribution of classical monocytes, we used tissue-protected BM chimeric mice (Fig. 3a)^23^ to fate map CCR2-dependent BM-derived cells without disrupting tissue homoeostasis. Wild-type (WT; CD45.1^+^CD45.2^+^) mice that had received hind leg irradiation were reconstituted with congenic (CD45.2^+/+^) WT BM, resulting in chimerism of between 20 and 30% donor origin in peripheral blood leukocytes, including Ly6C^+^ monocytes by 8 weeks post irradiation (Fig. 3b). This chimerism was mirrored in peritoneal Ly6C^+^ monocytes and all F4/80^lo^MHCII^+^ populations (Fig. 3c), confirming their replenishment by BM-derived precursors. Replenishment of peritoneal Ly6C^+^ monocytes and the CSF1R^+^CD11c^–^ fraction of F4/80^lo^MHCII^+^ cells was CCR2 dependent, as chimerism in these populations was absent in recipients of *Ccr2*^−/−^ BM (Fig. 3c). Furthermore, Ly6C^+^ monocytes likely utilize CCR2 to enter the peritoneal cavity, as the chimerism of these cells in the cavity was markedly lower than that of circulating Ly6C^+^ monocytes in *Ccr2*^−/−^>WT chimeras (Fig. 3a,b). Notably, CSF1R^−^CD11c^+^ cDC, granulocytes and B2 B cells were replenished equivalently or to a greater degree by *Ccr2*^−/−^ compared with WT BM (Fig. 3c). Importantly, although the F4/80^hi^ macrophage population remained predominantly of host origin 8 weeks after reconstitution, donor-derived cells could be identified in the recipients of WT BM, and this chimerism was virtually abolished in recipients of *Ccr2*^−/−^ BM (Fig. 3c) suggesting some Ly6C^+^ monocytes contribute to this population. Administration of anti-CCR2 mAb MC21 (ref. 32) at the time of transfer had no effect on donor chimerism in peritoneal F4/80^hi^ macrophages, ruling out the possibility that CCR2^+^ haematopoietic stem cells (HSC) present within the i.v. bolus are directly recruited to the cavity at the point of reconstitution (Fig. 3d).

CSF1R^+^CD11c^+^ cells among F4/80^lo^MHCII^+^ cells also exhibited reduced chimerism in mice engrafted with *Ccr2*^−/−^ marrow (Fig. 3c), however this was not absolute, suggesting that this population may be heterogeneous, a finding consistent with its partial reduction in *Flt3l*^−/−^ mice. Notably, CD64, a marker of macrophages in multiple tissues^10^,^14^,^27^,^33^, did not aid delineation of CSF1R^+^CD11c^+^ cells despite expression by F4/80^hi^ PEC cells and Ly6C^+^ monocytes (Fig. 3e). In contrast, analysis of whole transcriptome data from the Immunological Genome Consortium^14^ revealed a cluster of six highly differentially expressed genes in F4/80^lo^MHCII^+^CSF1R^+^ cells (both CD11c^+^ and CD11c^–^ fractions) (Fig. 3f) that included *Mrc1* and *Retnla,* which encode CD206 (mannose receptor) and the immunoregulatory cytokine RELMα, respectively. Flow cytometric analysis revealed RELMα to be largely expressed by F4/80^lo^MHCII^+^CSF1R^+^ cells but not Ly6C^+^ monocytes or CSF1R^–^CD11c^+^ cDC in the cavity (Fig. 3g). Importantly, RELMα cells amongst the CSF1R^+^CD11c^+^ subset showed high dependence on intrinsic expression of CCR2 (Fig. 3h). Thus, RELMα expression delineates a population of Ly6C^+^ monocyte-derived cells from RELMα^–^ DC amongst the CSF1R^+^CD11c^+^ subset. Expression of CD206 was less population restricted (Fig. 3g) and unable to fully delineate the CSF1R^+^CD11c^+^ subset (data not shown).

Thus, Ly6C^+^ monocytes continually extravasate into the peritoneal cavity via a CCR2-dependent mechanism to replenish CD11c^+^ and CD11c^–^ F4/80^lo^ macrophages and at least some acquire an F4/80^hi^ phenotype.

### Gradual replenishment of F4/80^hi^ macrophages by BM monocytes

**Figure 4:**
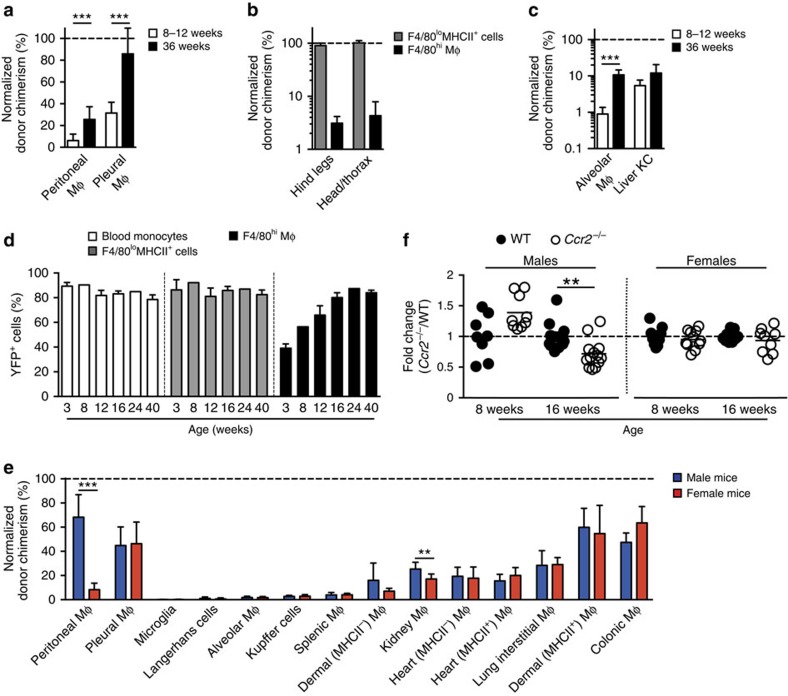
F4/80^hi^ Macrophages are slowly but continually replenished by monocytes. (**a**) Non-host chimerism of peritoneal F4/80^hi^ and F4/80^lo^MHCII^+^ cells from female tissue-protected BM chimeric mice normalized to Ly6C^+^ blood monocytes 8–12 (*n*=10) or 36 weeks (*n*=9) after reconstitution with WT BM. Bars represent the mean+s.d. and are pooled from two independent experiments. ***P<0.001 Student's *t*-test with Holm-Sidak correction. (**b**) Non-host chimerism amongst peritoneal F4/80^hi^ macrophages and F4/80^lo^MHCII^+^ subsets from WT>WT-protected BM chimeras in which the hind legs/lower abdomen or head and upper thorax was irradiated. Mice were analysed 8 weeks after reconstitution and chimerism was normalized to blood monocytes. Bars represent the mean+s.d. and are from one experiment with 5 mice per group. (**c**) Non-host chimerism of alveolar macrophages and Kupffer cells from female tissue-protected BM chimeric mice normalized to Ly6C^+^ blood monocytes 8–12 or 36 weeks after reconstitution with WT BM. Data are from one of two independent experiments performed with 5 mice per time point. ***P<0.001 Student's *t*-test with Holm-Sidak correction. (**d**) Expression of YFP by CSF1R^+^ blood monocytes, F4/80^lo^MHCII^+^ cells and F4/80^hi^ peritoneal macrophages from *Flt3*^Cre^.*Rosa26*^YFP^ mice at 3, 8, 12, 16, 24 and 40 weeks of age. Bars represent the mean+s.d. and are from one experiment with 2 (8,24 weeks) or 3 (3, 12, 16 and 40 weeks) mice. (**e**) Normalized non-host chimerism of peritoneal F4/80^hi^ macrophages and macrophages from the indicated organs (Supplementary Table 1) from male and female tissue-protected BM chimeric mice 9–11 weeks after reconstitution with WT sex-matched BM. Data are normalized to the non-host chimerism of Ly6C^+^ monocytes. **P<0.01, ***P<0.0001 Student's *t*-test with Holm-Sidak correction. Bars represent the mean+s.d. and are pooled from two experiments with 10 mice per group or from one experiment (splenic macrophages and Langerhans cells) with 5 mice per group. (**f**) Absolute numbers of PEC F4/80^hi^ macrophages from 8 week and 16 week old *Ccr2*^−/−^ mice, presented as a ratio to the number in age-matched WT mice (*Ccr2*^−/−^/WT). Symbols represent individual animals and horizontal bar is the mean. Data represent 8–13 mice per group pooled from two independent experiments. **P<0.005 Student's *t*-test with Holm-Sidak correction.

Given the contribution of Ly6C^+^ monocytes to the F4/80^hi^ macrophage population identified above, we next sought to determine if this contribution continued over time. Whereas only ∼8% of F4/80^hi^ macrophages derived from donor BM at 8–12 weeks post reconstitution, this increased significantly to ∼20% by 36 weeks (Fig. 4a), suggesting the autonomy of F4/80^hi^ macrophages does not persist over time. Unexpectedly, BM input occurred at a much greater rate and was maximal by 36 weeks in the pleural cavity, an anatomically similar site that harbours a phenotypically identical F4/80^hi^ macrophage population (Fig. 4a). Chimerism amongst F4/80^hi^ macrophages was not due to direct exposure of the cavities to radiation, as animals irradiated in different orientations displayed comparable degrees of chimerism amongst these cells (Fig. 4b), and consistent with fate mapping in *Flt3*^Cre^.*Rosa26*^YFP^ mice^7^, we also found progressive incorporation of BM-derived cells into the alveolar macrophage compartment over this 36-week period, whereas chimerism in liver Kupffer cells remained largely unchanged (Fig. 4c), suggesting these two techniques produce similar results. Nevertheless, we next performed a time course analysis of *Flt3*^Cre^.*Rosa26*^YFP^ mice, in which all HSC and their progeny are irreversibly labelled with YFP expression^4^. *Flt3*^Cre^.*Rosa26*^YFP^ mice faithfully labelled PEC of HSC origin, as all F4/80^lo^MHCII^+^ cells, which derive from BM, were labelled uniformly at all ages (Fig. 4d). In contrast, only ∼40–50% of the F4/80^hi^ macrophage population was labelled in juvenile (3-week old) and adult (8-week old) mice, but this increased progressively with age resulting in the entire F4/80^hi^ macrophage population becoming labelled by 24 weeks of age (Fig. 4d) and indicating that although non-HSC-derived macrophages exist in young mice, these are progressively displaced by HSC-derived cells with age.

Although both our tissue-protected BM chimera and *Flt3*-driven fate mapping techniques revealed progressive replenishment of peritoneal F4/80^hi^ macrophages by BM-derived cells, these methods suggested markedly different rates, with slower replenishment in the chimera system (∼0.5 and ∼2.7% change per week using BM chimeras and *Flt3*^Cre^.*Rosa26*^YFP^ mice, respectively). Notably, all our BM chimera studies utilized female mice, whereas *Flt3*^Cre^.*Rosa26*^YFP^ mice were male (due to the location of the transgene on the Y chromosome^7^). To assess whether gender influenced macrophage replenishment, we compared the acquisition of chimerism by tissue macrophages (Supplementary Table 1) from male and female tissue-protected BM chimeric mice (Fig. 4e). This revealed a striking difference in the rate of replenishment of PEC F4/80^hi^ macrophages, with more than sixfold greater replacement in male mice (Fig. 4e). Apart from kidney F4/80^hi^CD11b^lo^ macrophages, this gender difference did not appear to be present in any other tissue macrophage compartment examined, including the pleural cavity. Consistent with this gender difference, we found that although the absolute numbers of PEC F4/80^hi^ macrophages was equivalent in young WT and *Ccr2*^−/−^ mice, as previously reported^13^, CCR2 deficiency impacted the F4/80^hi^ macrophage compartment with age in male but not female mice (Fig. 4f). Thus, although seeded by embryo-derived macrophages, the source of the PEC F4/80^hi^ macrophage compartment changes with age and gender influences the rate of this change.

### BM-derived macrophages largely phenocopy embryo macrophages

**Figure 5:**
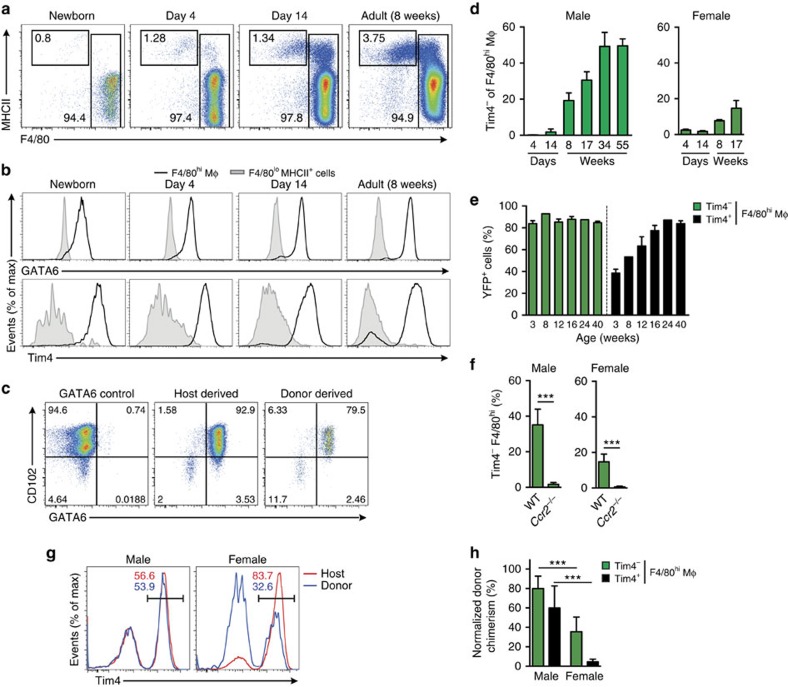
BM-derived F4/80^hi^ macrophages largely phenocopy embryonic macrophages. (**a**) Representative expression of F4/80 and MHCII by live CD45^+^Lin^−^ PEC from WT mice at d0-1 (newborn), days 4, 7, 14 and 28, and 8-week old adult mice. Data are from one experiment representative of five performed. (**b**) Representative expression of GATA6 and Tim4 by F4/80^hi^ macrophages and F4/80^lo^MHCII^+^ cells from peritoneum of WT mice as in **a**. (**c**) Expression of GATA6 and CD102 by host- and donor-derived peritoneal F4/80^hi^ macrophages from tissue-protected BM chimeric mice 36 weeks after reconstitution with WT BM. Representative flow plots from one experiment of two performed. (**d**) The mean frequency of Tim4^−^ cells amongst F4/80^hi^ macrophages obtained from male and female mice of the indicated ages. Bars represent the mean+s.d. with 3 (males; day 4 & 14), 4 (males; 8, 17, 34, 55 weeks) or 5 mice (all female time points). (**e**) Expression of YFP by Tim4^−^ and Tim4^+^ F4/80^hi^ macrophages from *Flt3*^Cre^.*Rosa26*^YFP^ mice at 3, 8, 12, 16, 24 and 40 weeks of age. Bars represent the mean+s.d. and are from one experiment with 2 (8 & 24 weeks) or 3 (3, 12, 16 and 40 weeks) mice. (**f**) The frequencies of Tim4^−^F4/80^hi^ macrophages and from the PEC of WT or *Ccr2*^−/−^ age-matched male mice. Bars represent the mean+s.d. and are pooled from two independent experiments with 6 (female) or 9 (male) mice per group). ***P<0.0001 determined by Student's *t*-test. (**g**) Expression of Tim4 by host- and donor-derived PEC F4/80^hi^ macrophages from male and female tissue-protected BM chimeric mice 9–11 weeks after reconstitution with WT BM. (**h**) Normalized non-host chimerism of peritoneal Tim4^−^ and Tim4^+^ F4/80^hi^ macrophages from male and female tissue-protected BM chimeric mice 9–11 weeks after reconstitution with WT sex-matched BM. Data are normalised to the non-host chimerism of Ly6C^+^ monocytes. ***P<0.001 determined by Student's *t*-test followed by Holm-Sidak correction. Bars represent the mean+s.d. and are pooled from two independent experiments with 10 mice per group.

We next sought to determine if BM-derived F4/80^hi^ macrophages adopt the characteristic features of the embryonic cells they replace. Notably, F4/80^hi^ macrophages dominated the peritoneal cavity from birth and exhibited a stable MHCII^lo^ phenotype throughout development (Fig. 5a). The transcription factor GATA6 is a defining feature of mature F4/80^hi^ PEC macrophages in adult mice^13^,^16^,^17^, and analysis of PEC from neonatal, adolescent and adult mice showed that F4/80^hi^ macrophages are GATA6^+^ throughout development (Fig. 5b). We exploited the low rate of chimerism in female mice to compare BM monocyte-derived and host-derived F4/80^hi^ macrophages, with the latter being predominated by embryo-derived cells. Importantly, BM-derived F4/80^hi^ macrophages adopted expression of GATA6, as well as CD102, a GATA6-independent feature of PEC F4/80^hi^ macrophages (Fig. 5c and Supplementary Fig. 2).

Expression of the phagocytic receptor Tim4 has also been used to define ‘resident' F4/80^hi^ macrophages among PEC^16^. Indeed, F4/80^hi^ macrophages from neonatal and juvenile mice were almost uniformly Tim4^+^, however, the F4/80^hi^ population in adult mice contained a significant Tim4^–^ fraction that accumulated with age (Fig. 5b,d). Because this occurred more quickly in male compared with female mice (Fig. 5d), we reasoned that Tim4^−^ macrophages could represent the appearance of BM-derived macrophages. Consistent with this idea, Tim4^−^F4/80^hi^ macrophages labelled uniformly in *Flt3*^Cre^.*Rosa26*^YFP^ mice at all time points (Fig. 5e) and were virtually absent from PEC of monocytopenic *Ccr2*^−/−^ mice (Fig. 5f). Importantly, however, BM-derived cells were able to acquire Tim4 expression in BM chimeric mice (Fig. 5g) and YFP^+^ cells accumulated in the Tim4^+^F4/80^hi^ PEC macrophage population with age. Finally, given the exclusive derivation of Tim4^−^F4/80^hi^ macrophages from BM, we used this marker to assess the longevity of BM-derived F4/80^hi^ macrophages in chimeric mice. Whereas >80% of Tim4^−^F4/80^hi^ macrophages had been replaced by BM in male mice by 10 weeks following reconstitution, this was significantly lower in female mice (∼40%) (Fig. 5h). This indicates that BM-derived macrophages that had entered the F4/80^hi^ macrophage compartment before irradiation still accounted for >60% of the Tim4^–^F4/80^hi^ macrophage population post reconstitution in female mice, thereby demonstrating that BM-derived macrophages become relatively long-lived. However, <5% of Tim4^+^ macrophages in female had been replaced, demonstrating a significantly greater half-life in this population. Collectively, these data demonstrate that BM-derived cells can become long-lived and acquire the cardinal features of the embryonically derived macrophages they replace, but that some features for example, Tim4 expression are not universally adopted by BM-derived cells.

### F4/80^lo^CSF1R^+^ cells are precursors of F4/80^hi^ macrophage**s**

**Figure 6:**
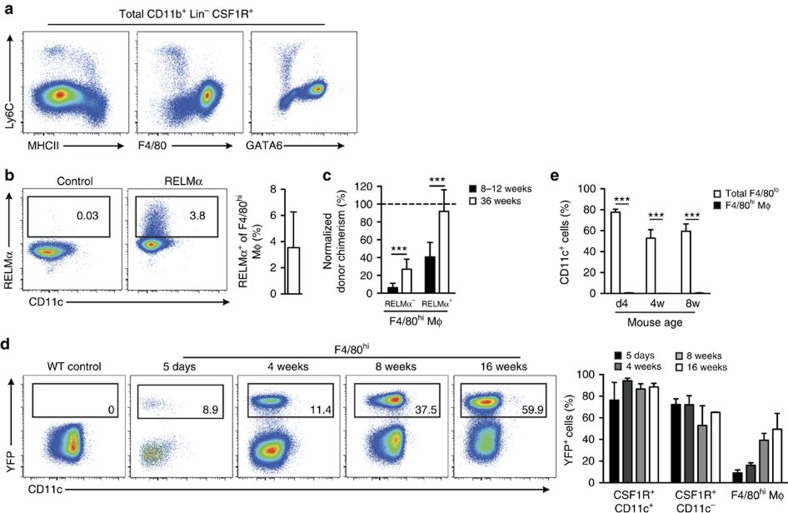
F4/80^lo^CSF1R^+^ cells are precursors of F4/80^hi^ macrophages. (**a**) Representative expression of Ly6C, MHCII, F4/80 and GATA6 by total CD11b^+^CSF1R^+^ cells obtained from the peritoneal cavity of 8-week old WT mice. (**b**) Representative expression of RELMα by F4/80^hi^ macrophages and mean frequency of RELMα^+^F4/80^hi^ macrophages among PEC of 8-week old WT mice. Data from one experiment of at least 10 performed. Bar represents the mean+s.d. with 5 mice. (**c**) Non-host chimerism of RELMα^+^ and RELMα^–^ PEC F4/80^hi^ macrophages from tissue-protected BM chimeric mice 8–12 or 36 weeks after reconstitution with WT BM. Data are normalized to the non-host chimerism of Ly6C^+^ blood monocytes. Bars represent the mean+s.d. and are pooled from two independent experiments with 10 mice per group. ***P<0.0001 determined by Student's *t*-test followed by Holm-Sidak correction. (**d**) Representative CD11c and YFP expression by F4/80^hi^ macrophages among PEC of *Itgax*^Cre^.*Rosa26*^YFP^ mice at the indicated ages. Right, mean frequencies of YFP^+^ cells amongst F4/80^hi^ macrophages, CSF1R^+^CD11c^−^ and CSF1R^–^CD11c^+^ cells. Bars represent the mean+s.d. and are from one experiment (d5, 4 weeks, 16 weeks) or pooled from two experiments (adult) with 2 (16 weeks), 4 (8 weeks), 7 (d5) or 8 mice (4 weeks). (**e**) Mean expression of CD11c by total F4/80^lo^MHCII^+^ cells and F4/80^hi^ macrophages from the peritoneal cavity of mice at 4 days, 4 weeks or 8 weeks of age. Bars represent the mean+s.d. and are from one of four experiments performed 5 mice per time point. ***P<0.0001 determined by Student's *t*-test followed by Holm-Sidak correction.

Ly6C^+^ monocytes have been shown to extravasate into a number of tissues in the steady state and mature through a so called ‘monocyte waterfall' to replenish tissue macrophages; a process which involves progressive acquisition of MHCII and loss of Ly6C^9^,^33^,^34^. We detected a similar process in the steady-state peritoneum. Notably, all Ly6C^+^ monocytes appeared to differentiate directly into F4/80^lo^MHCII^+^GATA6^−^ cells through a Ly6C^+^MHCII^+^ intermediary (Fig. 6a), suggesting that monocytes entering the F4/80^hi^ compartment may first transit through the F4/80^lo^MHCII^+^ phenotype. Consistent with this, a minor fraction of F4/80^hi^ macrophage cells expressed RELMα (Fig. 6b), a characteristic feature of monocyte-derived cells in the F4/80^lo^MHCII^+^ compartment. RELMα^+^F4/80^hi^ macrophages had a consistently higher level of non-host chimerism than the RELMα^−^ fraction in protected BM chimeric mice (Fig. 6c), suggesting a more recent BM origin and likely descent from F4/80^lo^MHCII^+^ cells. To directly map the role of F4/80^lo^CSF1R^+^ cells in generation of F4/80^hi^ macrophages, we utilized *Itgax*^Cre^.*Rosa26*^YFP^ mice, in which all F4/80^lo^MHCII^+^ cells show high degrees of YFP labelling throughout development (Fig. 6d). Importantly, YFP^+^ cells accumulated among F4/80^hi^ macrophages with age (Fig. 6d), despite this population remaining uniformly CD11c^−^ during this period (Fig. 6e). Thus, the presence of YFP^+^ cells in the F4/80^hi^ compartment indicates historical CD11c expression by their progenitors, suggesting that monocyte-derived cells actively transit through a CD11c^+^ stage en route to becoming F4/80^hi^ macrophages.

### Replacement of F4/80^hi^ cells is not due to exhaustion

**Figure 7:**
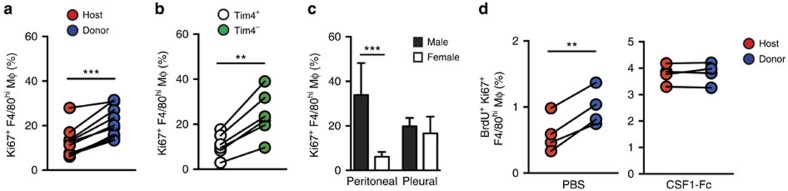
Replacement of F4/80^hi^ cells is not due to proliferative exhaustion. (**a**) Expression of Ki67 by host- and donor-derived PEC F4/80^hi^ macrophages from tissue-protected BM chimeric mice (8 weeks). Each symbol represents an individual animal and data are pooled from two independent experiments with 10 mice. ***P<0.0001 as determined by paired Student's *t*-test. (**b**) Expression of Ki67 by PEC Tim4^−/+^ F4/80^hi^ macrophages from 12-week old WT mice. Each symbol represents an individual animal and data are from one of three independent experiments with 6 mice. **P<0.005 as determined by paired Student's *t*-test. (**c**) Expression of Ki67 by peritoneal or pleural F4/80^hi^ macrophages from 6- to 8-week old WT male or female mice. Bars represent the mean+s.d. and data are pooled from two independent experiments with 8 (males) or 9 mice (females). ***P<0.0001 as determined by Student's *t*-test followed by Holm-Sidak correction. (**d**) Frequency of Ki67^+^ BrdU^+^ cells in host- and donor-derived peritoneal F4/80^hi^ macrophages from tissue-protected BM chimeric mice (12 weeks) following two doses of CSF1-Fc s.c. Each symbol represents an individual animal and data are from one of two independent experiments performed with 4 mice per group. **P<0.005 as determined by paired Student's *t*-test.

*In situ* proliferation is perceived as a property indicative of embryo-derived resident macrophages^35^,^36^. Furthermore, it has been proposed that proliferative exhaustion of embryonic macrophages explains their replacement in some tissues for example, the heart^12^. To test these theories in the peritoneal cavity, we first examined the rate of proliferation in donor and host-derived F4/80^hi^ macrophages from the peritoneum of protected BM chimeric mice. Notably, BM-derived donor F4/80^hi^ macrophages had a significantly higher rate of proliferation than their host counterparts (Fig. 7a). In addition, proliferation was significantly higher in Tim4^−^F4/80^hi^ macrophages compared with their Tim4^+^ equivalents in WT mice (Fig. 7b). Comparison of F4/80^hi^ macrophages from the peritoneal and pleural cavities of male and female mice revealed that those in the male PEC show the highest level of proliferation, consistent with the heightened proliferative activity of recent BM monocyte-derived macrophages (Fig. 7c). Thus, resident macrophages of recent BM origin exhibited the greatest proliferative activity in the cavities, a finding consistent with the non-stochastic mode of proliferation identified in our H2B-GFP pulse-chase studies. To determine whether the lower proliferative capacity of incumbent F4/80^hi^ macrophages is a result of exhaustion, we administered a CSF1-Fc fusion protein that induces extensive proliferation of resident macrophages^24^,^37^ to tissue-protected BM chimeric mice. Importantly, any difference in proliferative activity of host and donor cells was normalized by CSF1-Fc treatment, demonstrating that the incumbent embryo-derived macrophages did not have an inherent inability to proliferate (Fig. 7d). Taken together, these data indicate that *in situ* self-renewal during homoeostasis is not an exclusive property of embryonically derived macrophages, and that BM-derived cells eventually predominate the F4/80^hi^ macrophage compartment through a process of gradual displacement rather than local senescence. Thus, local tissue specific factors likely control macrophage longevity, the recruitment of monocytes and the imprinting of functional identity of macrophages regardless of origin (Supplementary Fig. 3).

## Discussion

Despite being one of the most studied populations of macrophages, the developmental origin of resident peritoneal macrophages remains contentious^5^,^6^,^25^. Here we show for the first time that although the peritoneal cavity is seeded initially by embryo-derived macrophages, BM-derived monocytes displace these in a process that is highly gender specific and not the result of proliferative exhaustion of incumbent embryo-derived population. We show that BM-derived macrophages largely phenocopy their embryo-derived counterparts and display extensive proliferative capacity, thus countering the widely held view that *in situ* self-renewal is synonymous with embryonic origin. Furthermore, we establish that F4/80^lo^MHCII^+^ (small) peritoneal macrophages act, at least in part, as intermediaries between newly arrived monocytes and mature F4/80^hi^ macrophages. Hence, unlike the divergent non-overlapping mechanisms currently perceived to control other tissue macrophages^5^,^6^,^7^,^9^,^10^,^11^,^12^,^25^,^38^,^39^,^40^, homoeostasis of resident peritoneal macrophages occurs neither exclusively by self-maintenance nor renewal from the BM, but through a combination of these processes.

Maintenance of tissue macrophage autonomy is proposed to rely on longevity and *in situ* self-renewal^41^. Our H2B-GFP pulse-chase system strongly suggests that all F4/80^hi^ macrophages in the peritoneum were capable of self-renewal, something not tested directly before due to limitations of traditional approaches for example, labelling with BrdU^42^. Furthermore, this revealed that proliferative heterogeneity exists, supporting a model of non-stochastic proliferation that is consistent with our observation that resident macrophages of recent monocyte origin exhibit higher levels of proliferation than those within the incumbent population. The lack of H2B-GFP label-retaining cells among F4/80^hi^ macrophages would also argue against the presence of quiescent progenitor cells akin to the near dormant HSC in bone marrow^31^ or a subset of senescent cells within this population implied from single-cell gene expression analyses of the response to CSF1 (ref. 30), while also arguing against the existence of a hierarchy of proliferating precursors and terminally differentiated daughter cells similar to that present amongst Langerhans cells in the epidermis^8^. Although we cannot exclude the possibility of non-proliferation-associated losses of GFP, confocal analysis revealed no evidence of extra-nuclear GFP in resident peritoneal cells (data not shown), and the rate of GFP loss was strongly associated with the frequency of Ki67^+^ macrophages within the population detected under different conditions (that is, in male versus female mice or after treatment with exogenous macrophage mitogens). Furthermore, our results are entirely consistent with the gradual loss of a membrane-associated dye within the entire resident population that has been reported previously^43^. Hence, while we cannot categorically rule out the possibility that macrophage progenitors exist within the peritoneal cavity, it is highly unlikely these are present among the F4/80^hi^ population, and although it is possible they display a distinct phenotype before maturing into F4/80^hi^ macrophages, akin to microglia progenitors^44^, our subsequent fate mapping studies would suggest that any local progenitor ultimately has only a limited input.

Our analysis of PEC F4/80^hi^ macrophages during development, combined with *Flt3*- and *Itgax-*driven fate mapping and BM chimeric mice clearly demonstrated a postnatal switch in the origin of peritoneal macrophages. Our results using *Flt3*^Cre^.*Rosa26*^YFP^ mice are entirely consistent with previous studies using these and other constitutive Cre systems (for example, *S100a4-*Cre and *Mx1-*Cre)^6^,^11^. However in those studies the significant but incomplete labelling at a single time point at adulthood was taken to support long-term persistence of embryonically derived macrophages in the peritoneal cavity^11^,^45^. In contrast, our time course analyses demonstrated a progressive change in macrophage origin and highlight the need for temporal analyses when using fate mapping systems. Parabiosis- and tamoxifen-inducible *Cx3cr1*^Cre/ER^.*Rosa26*^YFP^ mice have also been used to support the concept of autonomous peritoneal macrophages, but both may underestimate their contribution to tissue macrophages^46^, with parabiosis in particular having known limitations for determining fate of short-lived circulating precursors^47^.

In contrast to the studies of Hashimoto *et al.*^6^, Epelman *et al.*^11^ and Yona *et al.*^5^, recent *Kit*-driven fate mapping suggests that peritoneal F4/80^hi^ macrophages are exclusively of BM origin and have a turnover rate greater than that of colonic and dermal MHCII^+^ macrophages^25^, which rely on blood monocytes for their maintenance^9^,^10^,^34^. Sheng *et al.*^25^ proposed that mouse strain or microbiota in different animal facilities could underlie differences between studies, however, our data provides the novel explanation that gender is also a likely and previously unappreciated factor. While we would not rule out that the microbiological status of animals contributes to rate of macrophage turnover and hence differences between studies, elements of our studies were performed across multiple centres (see Methods section), yet each reached the same conclusion. Furthermore, it is unlikely that strain is the predominant factor underlying the differences between studies, as some of these used combinations of the same strains but arrived at opposing conclusions^6^,^25^,^43^. Thus we favour the idea that gender is a likely factor underlying some of the discrepancies between our study and others, and to the best of our knowledge, we are the first to assess the impact of gender on tissue macrophages dynamics using fate mapping approaches.

It remains unclear from our study why gender should influence the rate of macrophage displacement uniquely in the peritoneum, however the positioning of the female reproductive tract within the cavity likely underlies this phenomenon. Indeed, ovariectomy has been shown to alter the local chemokine milieu of the peritoneal cavity^48^ and peritoneal macrophages are known to express for example, oestrogen receptors^49^, allowing local hormones to influence macrophages directly^50^. In this respect, caution may need to be applied when using tamoxifen-based fate mapping due to the potential interference with ER signalling. Alternatively, given that adipokines are known to influence macrophage behaviour^51^, we cannot rule out that the increased abundance of perigonadal fat tissue in male abdomen may contribute to the sex difference in macrophage dynamics, particularly given the interplay between sex hormones and adiposity^52^. In addition, our data could indicate diverse gender-dependent utilization of peritoneal macrophages.

Consistent with numerous previous reports, we found that the numbers of F4/80^hi^ macrophages were unaffected by global *Ccr2* deficiency in young adult mice of either gender, a finding previously been interpreted to indicate their autonomy^13^,^53^, but that this changed with age, again highlighting the need for temporal analyses in studies of this nature. Together with data from our *Ccr2*^−/−^>WT-protected chimeric mice, which do not rely on whole-body irradiation as in other BM chimera approaches assessing CCR2 dependency^9^,^10^,^12^,^54^, we conclude that the BM-derived precursor responsible for replenishment is most likely Ly6C^+^ monocytes and that these cells are recruited to the steady-state cavities via a CCR2-dependent mechanism. These data would support reclassification of CCR2 as a primarily homoeostatic chemokine receptor rather than its current attribute as inflammatory one^55^ particularly given its redundancy in certain acute inflammatory settings^56^.

The distinct transcriptomic signature of F4/80^lo^MHCII^+^CSF1R^+^ peritoneal macrophages supports the idea of them performing unique functions from resident cells or monocytes. In particular production of the immunomodulatory cytokine RELMα and the c-type lectin CD206 was unique to these cells in the steady state inferring a role in the homoeostatic function of these cells. In addition, RELMα expression allowed delineation of CCR2-dependent cells among CD11c^+^CSF1R^+^ cells indicating it transiently marks cells of monocyte origin. Consistently, a small fraction of F4/80^hi^ macrophages expressed RELMα, and displayed more rapid replenishment from BM, suggesting that these are likely to be recent immigrants from the F4/80^lo^MHCII^+^ population. This combined with the progressive labelling in *Itgax*^Cre^.*Rosa26*^YFP^ mice, supports the idea of local maturation of F4/80^lo^MHCII^+^ macrophages into F4/80^hi^GATA6^+^ macrophages. While F4/80^lo^MHCII^+^ cells have been shown to differentiate into F4/80^hi^ macrophages, this was either following an inflammatory insult^5^ or in the absence of a full F4/80^hi^ macrophage niche^19^. Thus, we demonstrate for the first time that there is constitutive differentiation of Ly6C^+^ monocytes into F4/80^hi^ macrophages through a CD11c^+^ F4/80^lo^MHCII^+^ short-lived intermediate. Notably, the majority of CD11c^−^ F4/80^lo^ macrophages also appeared to derive from this CD11c^+^ intermediate.

Local environmental signals are responsible for the induction of specific macrophage signatures in different tissues^57^,^58^ and BM-derived F4/80^hi^ macrophages adopted GATA6 and CD102 expression; key signature characteristics of peritoneal macrophages. GATA6 is indispensible for development of F4/80^hi^ macrophages and F4/80^lo^ macrophages with high levels of MHCII, RELMα and CD206 accumulate in the peritoneum of mice with LysM-driven deletion of *Gata6*, suggesting a breakdown in local maturation of F4/80^lo^ cells in the absence of GATA6 (refs 16, 17). Notably, we also found that pleural F4/80^hi^ macrophages are GATA6^+^CD102^+^, suggesting the environmental signals responsible for initiating macrophage differentiation may be shared between serous cavities. Whether origin has subtle bearing on resident macrophage function remains to be fully determined, however, fewer macrophages of adult monocyte origin expressed Tim4, leading to a gender-dependent change in composition of resident cavity and suggesting embryo-derived macrophages and their monocyte-derived counterparts may have differing roles for example, in clearance of apoptotic cells, which could contribute to the excessive and inefficient inflammatory response mounted by male mice upon peritoneal infection^48^ and mirrored in Tim4-deficient animals^59^.

Although the contribution of BM monocytes to tissue macrophages is said to parallel reduction in proliferative activity of incumbent macrophages (for example, in the heart), proliferative exhaustion is unlikely to explain the initiation of monocyte entry and maturation, since both host and donor F4/80^hi^ macrophages expanded equally in response to CSF1-Fc. Thus, since steady-state proliferation is driven by CSF1 (refs 22, 24), newly recruited cells could be exposed to greater levels of CSF1R signalling by for example, trafficking via the CSF1-rich omentum^60^. Indeed omental factors including the vitamin A metabolite retinoic acid (RA) are reported to maintain the F4/80^hi^ macrophage population^13^. Notably, in the absence of vitamin A, peritoneal F4/80^hi^GATA6^+^ macrophages are lost progressively with age. While this could represent the constant requirement for RA to maintain F4/80^hi^ macrophages, in light of our findings, it may also indicate that RA is essential for the *in situ* maturation of F4/80^lo^MHCII^+^ cells, particularly as development/maintenance of F4/80^hi^ peritoneal macrophages is independent of RA until week 6 of age^13^. In support of this notion, F4/80^lo^ macrophages with high levels of MHCII and CCR2 accumulate in the peritoneum of vitamin A deprived mice^13^.

In summary, our results place the resident peritoneal macrophage firmly within the spectrum of tissue macrophages that are replaced with time by BM monocytes, but with kinetics that are overwhelmingly influenced by gender. These data are consistent with local environment rather than origin as the predominant factor controlling functional specialization of macrophages^57^ and concur with a recent report^39^ placing longevity within this list of locally controlled features, although the exact factors responsible for regulating macrophage turnover remain to be determined with certainty.

## Methods

### Animals and reagents

Wild-type C57BL/6JOlaHsd CD45.2^+^, congenic CD45.1^+^ CD45.2^+^, *Ccr2*^−/−^ (ref. 61) and *Rosa26-rtTA:Col1a1-tet-O-H2B-GFP* (ref. 31) mice were bred and maintained in specific pathogen-free facilities at the University of Edinburgh, UK. C57BL/6J (Crl) mice for comparison to *Ccr2*^−/−^ animals were purchased from Charles River, UK, while in three irradiation chimera studies, *Ccr2*^−/−^ mice used for BM were bred at the University of Glasgow, UK. *Itgax-*Cre mice^62^ were crossed with *Rosa26*-LSL-YFP mice (a gift from Dr. Megan Mcleod) and maintained at the University of Glasgow, UK. *Flt3l*^−/−^ mice^63^ were bred and maintained at the VIB Inflammation Research Center, University of Ghent. *Flt3*-Cre mice^64^ and *Rosa26*-LSL-YFP have been described previously^4^ and were bred and maintained at Centre for Molecular and Cellular Biology of Inflammation, King's College London, UK. Experimental mice were age and sex matched and aged between 6 and 12 weeks unless specified. H2B-GFP mice were used between 6 and 20 weeks of age. Experiments performed at UK establishments were permitted under licence by the UK Home Office, and were approved by the University of Edinburgh Animal Welfare and Ethical Review Body, the University of Glasgow Local Ethical Review Panel or the Kings College Biomedical & Health Sciences, Dentistry, Medicine and Natural & Mathematical Sciences Research Ethics Subcommittee. Experiments performed at the University of Ghent were carried out in accordance with the Ethical Committee University of Ghent-Faculty of Science VIB. IL-4–anti–IL-4 mAb complex (IL-4c) and CSF1-Fc was prepared as described previously 24 and mice were injected s.c. with 0.25 μg g^−1^ bodyweight of recombinant IL-4 (PeproTech) complexed to 1.25 μg g^−1^ 11B11 (Bio X Cell), 1 μg g^−1^ CSF1-Fc, or 100 μl PBS vehicle at the indicated time points.

### Tissue-protected BM chimeric mice

Anaesthetized 6–8-week old C57BL/6J CD45.1^+^CD45.2^+^ animals were exposed to a single dose of 9.5 Gy γ-irradiation while all but the hind legs and lower abdomen, or where specified, all but the head and upper thorax of the animals, were protected by a 2 inch lead shield. Animals were subsequently given 2-5 × 10^6^ BM cells from CD45.2^+^ C57BL/6J or *Ccr2*^−/−^ animals by iv injection. In certain experiments, BM cells were given together with 20 μg of anti-CCR2 mAb (MC-21)^32^ or rat IgG2b control mAb (BioXcell, UK), or with PBS alone, and efficacy of anti-CCR2 mAb treatment determined by FACS analysis on 10 μl tail venous blood samples 24 h later. Unless specified, mice were left for a period of 8 weeks before analysis of chimerism in the tissue compartments.

### BrdU injection

For labelling of proliferating cells, mice were injected s.c. with 100μl of 10 mg ml^−1^ BrdU in Dulbecco's PBS 2 h before experimental end-point.

### Doxycycline administration

To induce H2B-GFP expression in H2B-GFP mice, mice were fed doxycycline-supplemented diet (IPS Product Supplies, UK) for 2 weeks. Mice were screened on the day of doxycycline withdrawal and mice with <95% labelling of blood monocytes were excluded from analysis.

### Preparation of single-cell suspensions

Mice were killed by exsanguination via the brachial artery under terminal anaesthesia and 20 μl of blood was mixed with Hanks's buffered saline solution containing 2 mM EDTA and reserved for haematological analysis. Peritoneal and pleural cavities were lavaged with RPMI containing 2 mM EDTA (Invitrogen). Any samples heavily contaminated with erythrocytes were excluded from analysis. Lungs, heart, kidney and brain were removed from perfused mice, chopped finely and digested in pre-warmed collagenase ‘cocktail' (0.625 mg ml^−1^ collagenase D (Roche), 0.85 mg ml^−1^ collagenase V (Sigma-Aldrich), 1 mg ml^−1^ dispase (Gibco, Invitrogen), and 30 U ml^−1^ DNase (Roche Diagnostics GmbH) in RPMI-1640) for 45 min in a shaking incubator at 37 °C before being passed through an 100 μm filter. Tissue preparations were washed in FACS buffer followed by centrifugation at 300*g* for 5 min. Erythrocytes were lysed using red blood cell lysis buffer (Sigma-Aldrich). Livers were removed from perfused mice, chopped finely and digested in pre-warmed collagenase ‘cocktail' (5 ml per liver) for 30 min in a shaking incubator at 37 °C before being passed through an 100 μm filter. Cells were washed twice in 50 ml ice cold RPMI followed by centrifugation at 300*g* for 5 min. Supernatants were discarded and erythrocytes were lysed. Colonic lamina propria, epidermal and dermal leukocytes were isolated as described previously^65^,^66^. The resulting cell suspension was subsequently passed through a 40 μm strainer before cell counting. All cells were maintained on ice until further use. Cellular content of the preparations was assessed by cell counting using a Casey TT counter (Roche) in combination with multi-colour flow cytometry.

### Discrimination of vascular and tissue-derived cells

According to published methods^28^, mice were injected i.v. with 1 μg FITC-conjugated anti-CD45.2 antibody and 5 U heparin sulphate in 100 μl DPBS 2 min before sacrifice (equivalent data was obtained with PE or PE-Cy7-conjugated antibodies). Control animals were injected with PBS alone.

### Flow cytometry

Equal numbers of cells or equivalent volumes of blood were stained with LIVE/DEAD (Invitrogen) or 7AAD (Biolegend), blocked with 0.025 μg anti-CD16/32 (2.4G2; Biolegend) and 1:20 heat-inactivated mouse serum (Invitrogen), and then stained with a combination of antibodies detailed in Supplementary Table 2. Where applicable, cells were subsequently stained with streptavidin-conjugated fluorochromes. Fluorescence-minus-one controls confirmed gating strategies, while discrete populations within lineage^+^ cells were confirmed by omission of the corresponding population-specific antibody. Erythrocytes in blood samples were lysed using FACS Lyse Solution (BD Biosciences).

For intracellular staining, cells were subsequently fixed and permeabilized using FoxP3/Transcription Factor Staining Buffer Set (eBioscience), and intracellular staining performed using antibodies detailed in Supplementary Table 2. For the detection of BrdU, cells were fixed as above and incubated with 3 μg DNaseI (Sigma) for 30–60 min, before being washed in PermWash (eBioscience) and then incubated with anti-BrdU antibody for 30 min at room temperature. Samples were acquired using FACS LSRFortessa using FACSDiva software (BD) and analysed with FlowJo version 9 software (Tree Star). Analysis was performed on single live cells determined using forward scatter height versus area and negativity for live/dead. For analysis of macrophage proliferation, Ki67 expression was used to determine the frequency of all F4/80^hi^ cells in cycle, whereas a 2 h BrdU pulse before necropsy combined with Ki67 expression was used to identify cells in S phase, as described previously^24^.

### Morphological assessment of cells

Peritoneal myeloid subsets as in Fig. 1b were FACS-purified using a FACSAriaII. Sorted cells were spun onto poly-L-lysine-coated glass microscope slides (VWR International), fixed in acetone and stained using the Rapid-Romanowsky staining kit (Raymond A. Lamb, Eastbourne, UK).

### Analysis of microarray data

Microarray data of different myeloid populations produced by the ImmGen consortium (GEO15907) was downloaded from Gene Expression Omnibus. Details of the sorting strategies used to isolate each population can be found at http://www.immgen.org. Raw data was normalized using the Robust Multi Array algorithm and a common threshold for positive expression was determined. All arrays passed visual interrogation of MA plots. Differential gene expression between populations was identified using the Affy^67^ and Limma^68^ bioconductor packages in R. Differentially expressed genes were determined using a minimum of a two-fold gene expression and adjusted *P* value of <0.01 (*t*-test) on Log_2_ transformed data. Similarity between populations was determined by hierarchical clustering of expressed genes using the Pearson's correlation coefficient generated on Log_2_ transformed data with the CBA and/or Affy package in R. Unsupervised K means clustering produced similar data to hierarchical clustering.

### Statistics

Where required, data were log transformed to achieve normal distribution and equal variance. One-way ANOVA was used to compare all means within a data set, whereas the Holm-Sidak correction was used when individual *t-*tests were performed on multiple cell populations. Paired Student's *t-*test was used to determine differences in frequency of Ki67^+^ cells within donor and host-derived macrophages obtained from tissue-protected BM chimeric mice, whereas unpaired Student's *t-*test was used for all other two sample analysis. Statistics were performed using Prism 6 (GraphPad Software). The statistical test used in each experiment is detailed in the relevant figure legend.

### Data availability

Data that support the findings of this study are available in Gene Expression Omnibus with the accession code GEO15907. All other data that support the findings of this study are available from the corresponding author upon request.

## Additional information

**How to cite this article:** Bain, C.C. *et al.* Long-lived self-renewing bone marrow-derived macrophages displace embryo-derived cells to inhabit adult serous cavities. *Nat. Commun.* 7:11852 doi: 10.1038/ncomms11852 (2016).

## Supplementary Material

Supplementary Information Supplementary Figures 1-3 and Supplementary Tables 1 & 2.

Peer Review File
