# Supplementary material for: Long-lived self-renewing bone marrow-derived macrophages displace embryo-derived cells to inhabit adult serous cavities
Source: Nat Commun. 2016 Jun 13;7:ncomms11852. doi: 10.1038/ncomms11852 (PMC4910019; doi:10.1038/ncomms11852)
Supplement: Supplementary Information — Supplementary Figures 1-3 and Supplementary Tables 1 & 2. [file ncomms11852-s1.pdf]

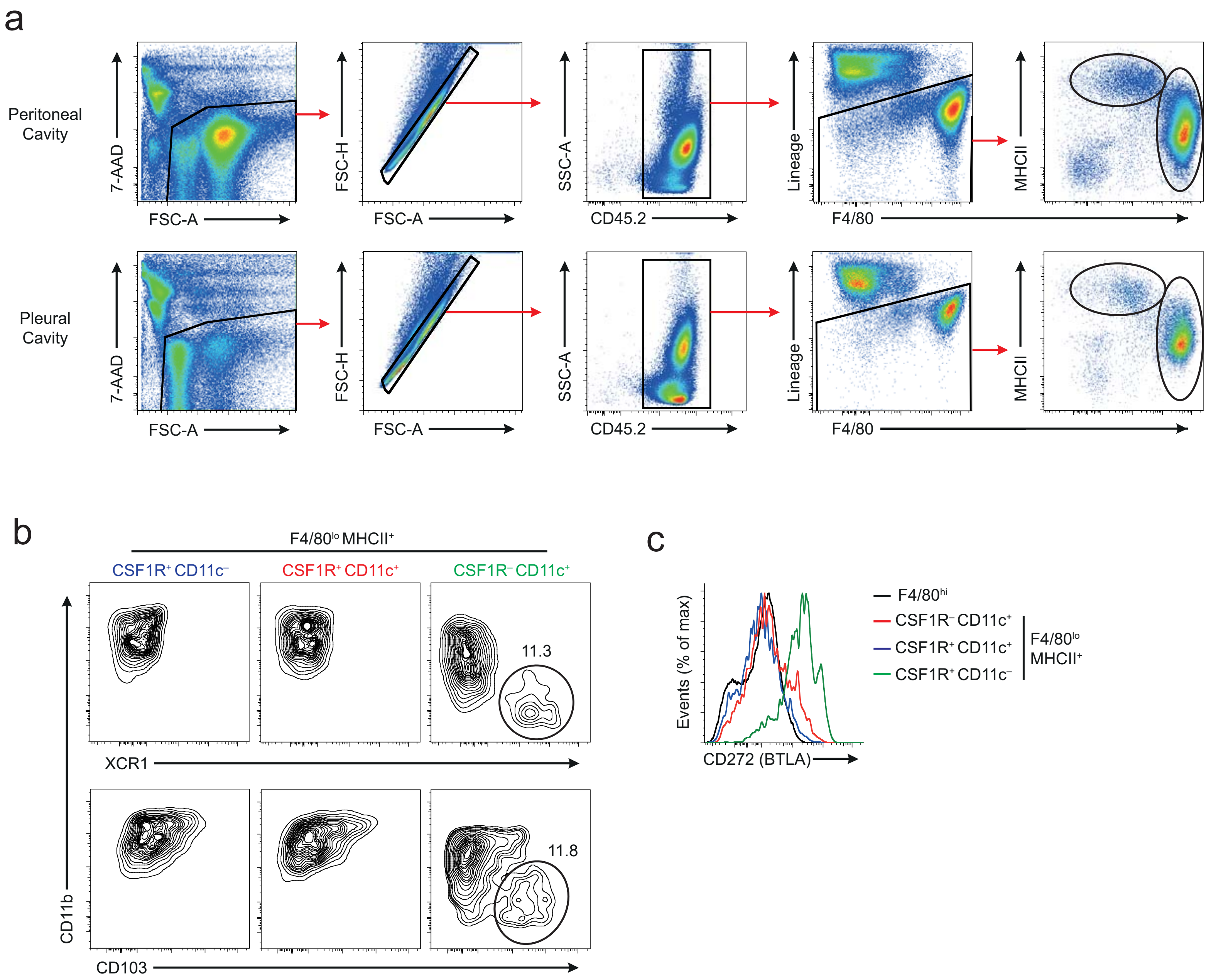

Supplementary Figure 1: Gating Strategy and Additional Phenotyping

(**a**) Gating strategy used to define F4/80^{hi} macrophages and F4/80^{lo}MHCII^{+} cells in the peritoneal and pleural cavities. (**b**) Representative expression of CD11b, CD103 and XCR1 by CSF1R^{+}CD11c^{-}, CSF1R^{+}CD11c^{+} and CSF1R^{-}CD11c^{+} F4/80^{lo}MHCII^{+} cells from peritoneal cavity of naïve WT mice. (**c**) Representative expression of CD272 (BTLA) by F4/80^{hi} macrophages and CSF1R^{+}CD11c^{-}, CSF1R^{+}CD11c^{+} and CSF1R^{-}CD11c^{+} F4/80^{lo}MHCII^{+} cells from peritoneal cavity of naïve WT mice. Data from **b** and **c** are representative of two independent experiments.

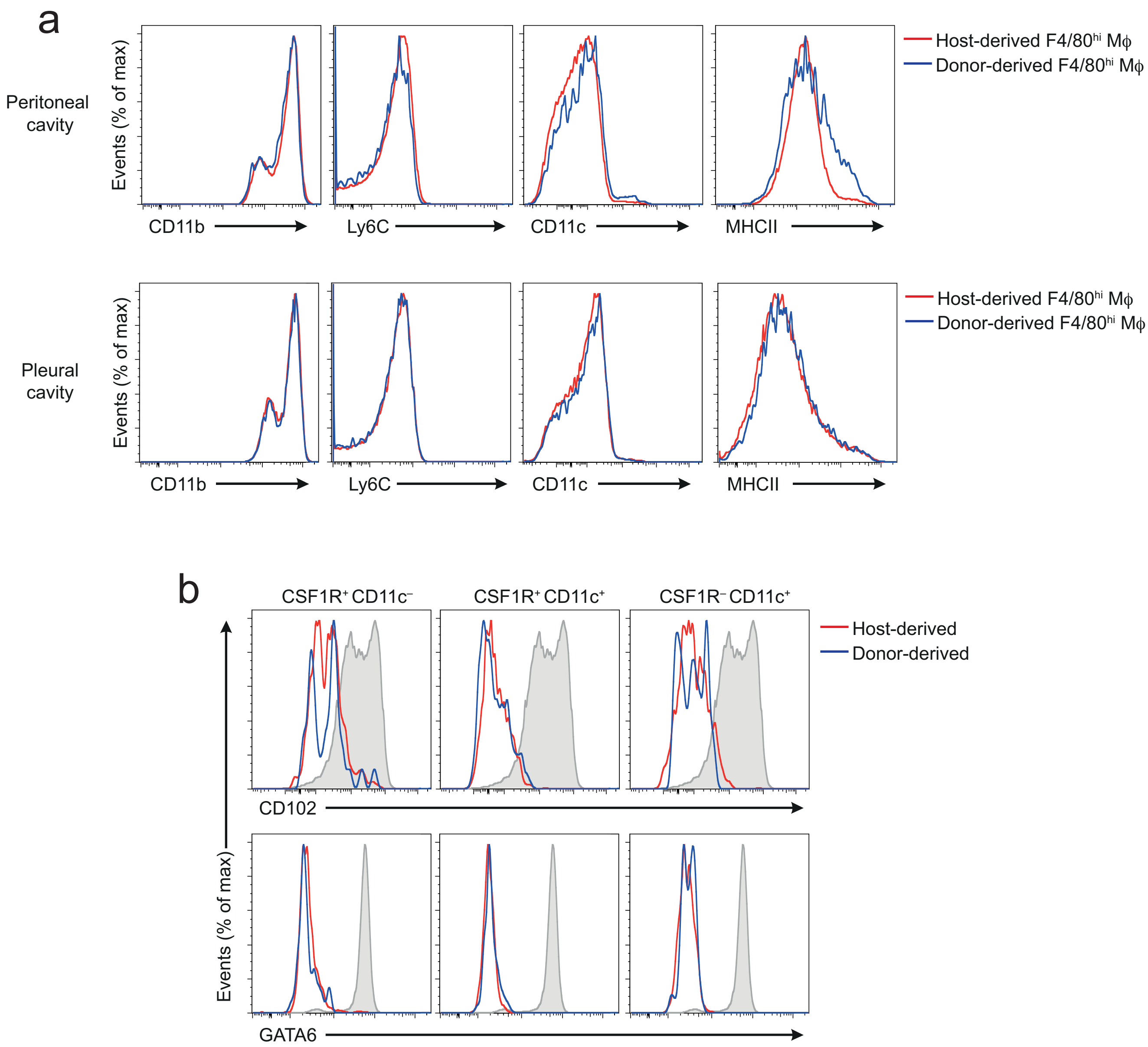

Supplementary Figure 2: Donor BM-Derived Macrophages Phenocopy Their Host-derived Equivalents

(**a**) Expression of CD11b, CSF1R, Ly6C, CD11c and MHCII by host- and donor-derived F4/80^{hi} macrophages from peritoneal and pleural cavities of tissue-protected BM chimeric mice 36 weeks after reconstitution with WT BM. Representative flow plots from one experiment of 2 experiments performed, with n=5 mice. (**b**) Expression of CD102 and GATA6 by host- and donor-derived CD11c/CSF1R-defined F4/80^{lo}MHCII^{+} subsets from the same mice as **a.** Shaded grey histograms represent expression by F4/80^{hi} macrophages.

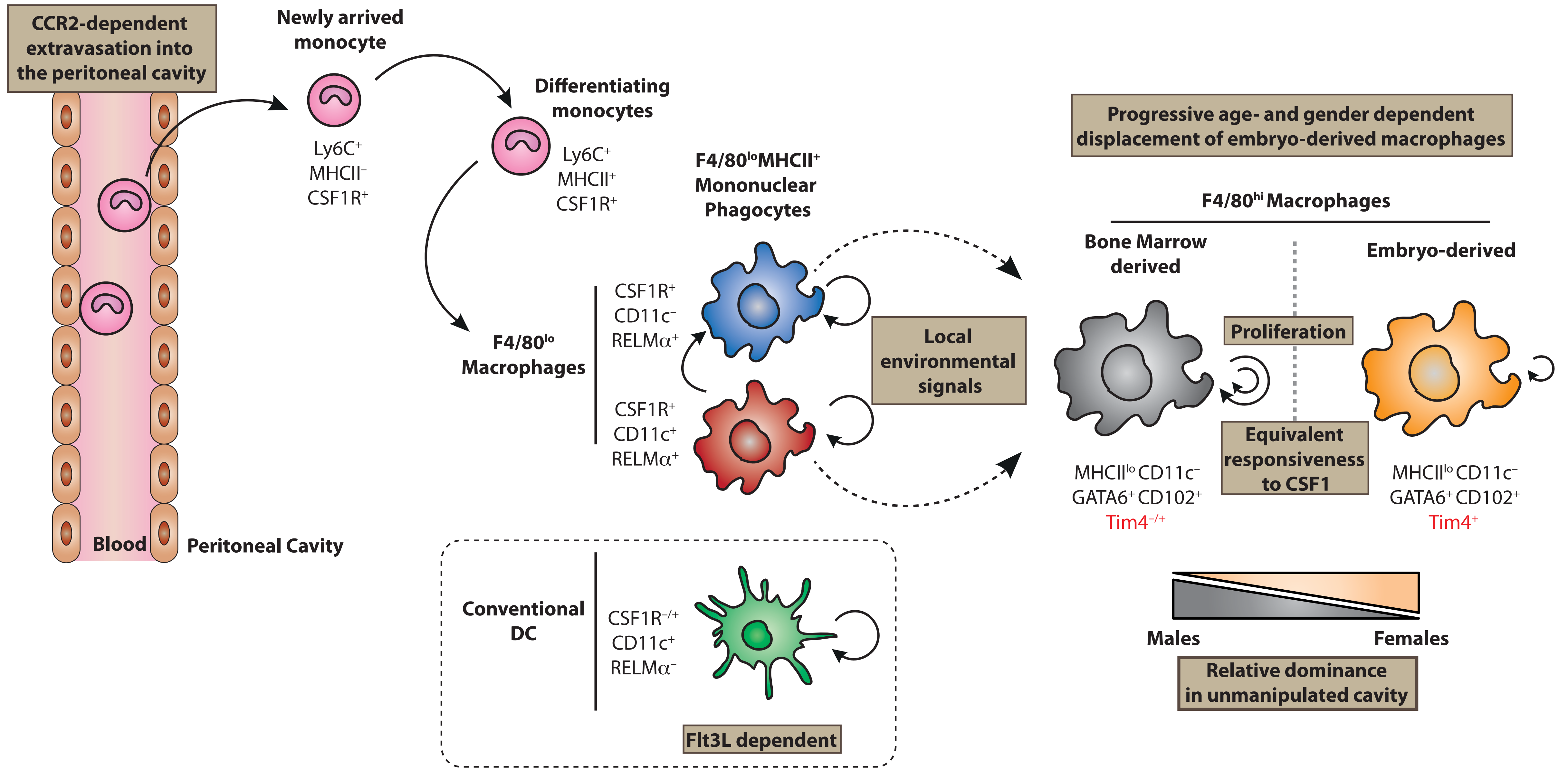

Supplementary Figure 3: Proposed Model of Peritoneal Macrophage Maintenance under Steady State Conditions

During development the peritoneal cavity is seeded by embryo-derived F4/80^{hi} macrophages (orange cells) that persist into adulthood, however the number of these declines with age. Circulating Ly6C^{+} blood monocytes continually extravasate into the peritoneal cavity in a CCR2-dependent process and mature through short-lived intermediates to replenish F4/80^{lo}MHCII^{+} macrophages. Maturation through this 'monocyte waterfall' involves acquisition of MHCII, CD11c and RELMα expression, and downregulation of Ly6C. These F4/80^{lo}MHCII^{+}CSF1R^{+}RELMα^{+} cells (red and blue cells) proliferate in situ and at least some mature further into F4/80^{hi}MHCII^{lo} 'resident' macrophages (black cells) in a process that is controlled by the local environment. F4/80^{lo}MHCII^{+}CSF1R^{-}RELMα^{-}CD11c^{+} (green cells) represent bona fide conventional dendritic cells and are not derived from Ly6C^{+} monocytes. BM-derived F4/80^{hi} macrophages largely phenocopy the embryo-derived cells they replace, although certain characteristics, such as expression of Tim4 and the rate of proliferation differs between macrophages derived from BM versus embryonic sources. Notably, the rate of displacement of embryo-derived F4/80^{hi} macrophages by BM-derived cells is highly gender specific, with more rapid replacement in males compared with female mice, supporting the notion that environmental signals control this process.

Supplementary Table 1: Phenotype of Tissue Macrophages (relates to Figure 4)

**Macrophage Population**	**Phenotype**
Peritoneal	Live $CD45^+$ $CD3^-$ $CD19^-$ $Ly6G^-$ $SiglecF^-$ $F4/80^{hi}$ $MHCII^{-/lo}$
Pleural	Live $CD45^+$ $CD3^-$ $CD19^-$ $Ly6G^-$ $SiglecF^-$ $F4/80^{hi}$ $MHCII^{-/lo}$
Microglia	Live $CD45^+$ $CD3^-$ $CD19^-$ $Ly6G^-$ $SiglecF^-$ $Ly6C^-$ $F4/80^{lo}$ $CD11b^+$ $MHCII^-$
Langerhans cells	Live $CD45^+$ $CD3^-$ $CD19^-$ $Ly6G^-$ $SiglecF^-$ $Ly6C^-$ $CD11c^+$ $MHCII^+$ $CD11b^+$ $EpCAM^+$
Alveolar Mϕ	Live $CD45^+$ $CD3^-$ $CD19^-$ $Ly6G^-$ $CD64^+$ $CD11c^{hi}$ $SiglecF^+$ $CD11b^{lo}$
Kupffer cells	Live $CD45^+$ $CD3^-$ $CD19^-$ $Ly6G^-$ $SiglecF^-$ $F4/80^{hi}$ $CD11b^{lo}$
Splenic Mϕ	Live $CD45^+$ $CD3^-$ $CD19^-$ $Ly6G^-$ $SiglecF^-$ non-$CD11c^{hi}$ $F4/80^{hi}$ $CD11b^{lo}$
Dermal Mϕ	Live $CD45^+$ $CD3^-$ $CD19^-$ $Ly6G^-$ $SiglecF^-$ $CD11b^+$ $CD24^-$ $CD64^{lo/+}$ $Ly6C^-$ then $MHCII^-$ or $MHCII^+$
Kidney Mϕ	Live $CD45^+$ $CD3^-$ $CD19^-$ $Ly6G^-$ $SiglecF^-$ $F4/80^{hi}$ $CD11b^{lo}$
Heart Mϕ	Live $CD45^+$ $CD3^-$ $CD19^-$ $Ly6G^-$ $SiglecF^-$ $F4/80^+$ $CD11b^+$ then $MHCII^-$ or $MHCII^+$
Lung interstitial Mϕ	Live $CD45^+$ $CD3^-$ $CD19^-$ $Ly6G^-$ $CD64^+$ $CD11c^{lo/+}$ $SiglecF^-$ $CD11b^+$ $Ly6C^-$ $MHCII^+$
Colonic Mϕ	Live $CD45^+$ $CD64^+$ $Ly6C^-$ $MHCII^+$ $CD11c^{lo/+}$

Supplementary Table 2: List of Antibodies

Antibody	Clone	Source	Dilution
**Mouse**			
BrdU	Bu20a	Biolegend	1/16
CD3	17A2	Biolegend	1/200
CD11b	M1/70	Biolegend	1/200-800*
CD11c	N418	Biolegend	1/200
CD16/32	2.4G2	Biolegend	1/200
CD19	6D5	Biolegend	1/200
CD45.1	A20	Biolegend	1/200
CD45.2	104	Biolegend	1/200
CD64	X54-5/7	Biolegend	1/200
CD102	3C4	Biolegend	1/200
CD103	2E7	eBioscience	1/200
CD115	AFS98	Biolegend	1/200
CD206	C068C2	Biolegend	1/400
CD272	6F7	Biolegend	1/200
CCR2	475301	R&D Systems	1/10
GATA6	D61E4	Cell Signalling Technologies	1/100
F4/80	BM8	Biolegend	1/200
Ki67	B56	BD Biosciences	1/4
Ly6C	HK1.4	Biolegend/eBioscience	1/400
Ly6G	1A8	Biolegend	1/200
MHC II (IA-IE)	M5/114.15.2	Biolegend/eBioscience	1/400
RELMα (purified)	Polyclonal	Peprotech	1/100
SiglecF	E50-2440	BD Biosciences	1/200
SiglecF	ES22-10D8	Miltenyi Biotec	1/28
Tim4	F31-5G3	Biolegend	1/2000
XCR1	ZET	Biolegend	1/200
Zenon anti-rabbit reagent		Molecular Probes	1/300
Streptavidin-BV650		BD Biosciences	1/200

* anti-CD11b was used at 1/200, 1/400 or 1/800 dependent on tissue and conjugate.
