# Supplementary material for: Long-lived self-renewing bone marrow-derived macrophages displace embryo-derived cells to inhabit adult serous cavities
Source: Nat Commun. 2016 Jun 13;7:ncomms11852. doi: 10.1038/ncomms11852 (PMC4910019; doi:10.1038/ncomms11852)
Supplement: Peer Review File [file ncomms11852-s2.pdf]

Editorial Note: this manuscript has been previously reviewed at another journal that is not operating a transparent peer review scheme. This document only contains reviewer comments and rebuttal letters for versions considered at Nature Communications.
